# Autoantibody Positivity in Bronchiectasis Without Interstitial Lung Diseases: Risk Factors and Impact on Clinical Features

**DOI:** 10.3390/jcm15082897

**Published:** 2026-04-10

**Authors:** Shuqiao Yang, Wanlu Sun, Li Gu

**Affiliations:** 1 Department of Infectious Diseases and Clinical Microbiology, Beijing Institute of Respiratory Medicine, Beijing Chao-Yang Hospital, Capital Medical University, Beijing 100020, China; shuqiao1987@mail.ccmu.edu.cn; 2 Department of Respiratory and Critical Care Medicine, Beijing Institute of Respiratory Medicine, Beijing Chao-Yang Hospital, Capital Medical University, Beijing 100020, China

**Keywords:** autoantibody, connective tissue disease, bronchiectasis, risk factor

## Abstract

**Objective:** To characterise circulating autoantibodies, identify associated risk factors, and evaluate their impact on clinical features in bronchiectasis (BR) patients without interstitial lung diseases (ILDs). **Methods:** This retrospective observational study analysed 506 non-ILD BR inpatients, categorised into isolated BR (*n* = 240), autoantibody (AAb)-positive BR (*n* = 131), and connective tissue disease (CTD)-BR (*n* = 135) groups. Clinical features were compared, and multinomial logistic regression was used to identify factors associated with AAb positivity and CTD. **Results:** Compared with isolated BR, older age (OR 1.062, 95% CI 1.022–1.103, *p* = 0.002) and prior PTB (OR 3.481, 95% CI 1.239–9.779, *p* = 0.018) were independently associated with AAb-positive BR, while CTD-BR was further associated with female sex (OR 2.677, 95% CI 1.021–5.586, *p* = 0.046) and greater lower-lobe involvement (OR 4.115, 95% CI 1.947–13.672, *p* = 0.015). A significantly higher frequency of *Pseudomonas aeruginosa* in the lower respiratory tract was observed in RA (rheumatoid arthritis)-BR compared to isolated BR, AAb-positive BR, and other CTD-BR (43.4% vs. 22.2%, 25.2%, and 18.2%, *p* = 0.002). This association remained significant after adjusting for confounders (OR 4.386, 95% CI 1.729–15.328, *p* = 0.007). **Conclusions:** In non-ILD BR patients, AAb positivity presented limited clinical differences compared with isolated BR, with older age and prior PTB independently associated, possibly reflecting underlying immune dysregulation. RA-BR showed a potential independent association with *P. aeruginosa* isolation, warranting further investigation.

## 1. Introduction

Bronchiectasis (BR) is a chronic respiratory disease characterised by irreversible dilation of the bronchi and recurrent infections. Patients with BR commonly present with chronic cough, sputum production, dyspnoea, and haemoptysis [1]. Over recent decades, there has been a noticeable rise in the global incidence and prevalence of BR, placing considerable economic strain on healthcare systems and causing a marked decline in patients’ quality of life [2].

The relationship between autoimmune disorders and BR has been increasingly recognised. In patients with connective tissue diseases (CTDs), BR may occur as an isolated condition or as a secondary manifestation of traction bronchiolitis in interstitial lung disease (ILD). Rheumatoid arthritis (RA) is the most frequently reported CTD type associated with BR [3]. Beyond RA, BR has also been reported as a pulmonary manifestation of other CTDs, including anti-neutrophil cytoplasmic antibody (ANCA)-associated vasculitis (AAV) [4]. It appears that BR is more strongly linked to specific autoantibodies. Dinache et al. demonstrated that RA patients positive for anti-cyclic citrullinated peptide (CCP) antibodies had a higher prevalence of BR [5]. Similarly, a recent meta-analysis revealed that BR was significantly more prevalent in myeloperoxidase (MPO)-ANCA-positive patients compared to those with proteinase 3 (PR3)-ANCA [6]. Beyond CTDs, BR has also been described in association with other systemic inflammatory conditions characterised by autoantibody (AAb) positivity, such as inflammatory bowel disease [7]. These observations suggest that BR may arise in broader contexts of immune dysregulation.

Autoantibodies may have important implications for the aetiological assessment and clinical management of BR. However, their prevalence and clinical relevance in BR, particularly among patients without ILDs, remain poorly defined. This study aimed to characterise circulating autoantibodies, identify associated risk factors, and evaluate their impact on clinical characteristics in BR patients without ILDs.

## 2. Methods

This retrospective observational study was conducted with approval from the Ethics Committee of Beijing Chao-Yang Hospital (approval No. 2025-ke-634). Given the use of existing medical records and the retrospective design, informed consent was waived.

### 2.1. Study Design and Population

Clinical data of inpatients diagnosed with BR between 2017 and 2023 were retrospectively extracted from the big data analytics platform jointly developed by the Beijing Institute of Respiratory Medicine and Beijing Chao-Yang Hospital. Patients meeting the diagnostic criteria for BR according to the British Thoracic Society Guidelines were included [8]. Exclusion criteria were: (1) absence or incomplete serological AAb testing; (2) active malignancy; (3) acute exacerbation at the time of sampling; or (4) chest CT unavailable.

Patients were categorised into three groups based on the presence of circulating autoantibodies and clinical diagnosis of CTDs: isolated BR, AAb-positive BR, and CTD-BR. CTDs were diagnosed according to established classification criteria [9,10,11,12]. AAb-positive BR was defined as the presence of circulating autoantibodies in patients who did not meet the diagnostic criteria for any CTD, as determined by comprehensive clinical evaluation, serological testing, and imaging. All diagnoses and classifications were independently reviewed and confirmed by an experienced rheumatologist to ensure diagnostic accuracy.

### 2.2. Data Extraction and Imaging Evaluation

Circulating autoantibodies, including antinuclear antibodies (ANA), anti-double-stranded DNA (dsDNA), anti-extractable nuclear antigens (ENAs), anti-CCP, ANCA, and rheumatoid factor (RF), were collected. ANA was assessed by indirect immunofluorescence, with titres ≥ 1:320 considered high. Anti-dsDNA, ENAs, CCP, ANCA, and RF were measured using standard immunoblotting or ELISA methods; high RF titres were defined as ≥2× the upper limit of normal (ULN).

The following information was also collected upon patients’ admission: (1) demographic details, comorbidities, and symptoms; (2) microorganisms detected in the lower respiratory tract (LRT); (3) spirometry parameters, including the percentages (%) of the predicted normal values for pre-bronchodilator forced expiratory volume in the first second (FEV1), forced vital capacity (FVC), single-breath diffusing capacity of the lung for carbon monoxide (DLCO_SB_), and the FEV1/FVC ratio; (4) disease severity, as evaluated by the Bronchiectasis Severity Index (BSI) [13]; (5) treatments, including inhaled bronchodilators, oral corticosteroids, disease-modifying antirheumatic drugs (DMARDs) and long-term (≥3 months) antibiotics (including macrolides).

All patients underwent chest CT. Affected pulmonary lobes were independently assessed by two experienced pulmonologists blinded to clinical data, with discrepancies resolved by consensus.

### 2.3. Statistical Analysis

Statistical analyses were performed using IBM SPSS Statistics, version 23 (IBM Corp., Armonk, NY, USA). Categorical variables were presented as frequencies and percentages, while continuous variables were reported as mean ± standard deviation (SD) or median (interquartile range, IQR), depending on distribution. Intergroup comparisons of continuous variables were conducted using one-way analysis of variance (ANOVA) or the Kruskal–Wallis test. Categorical variables were compared using the chi-square test. Spearman’s rank correlation coefficient (*r*) was used to assess correlations. Risk factors were evaluated using binary and multinomial logistic regression models as appropriate, with results expressed as odds ratios (ORs) and 95% confidence intervals (CIs). A *p*-value < 0.05 was considered statistically significant.

## 3. Results

### 3.1. Study Patients

**Figure 1:**
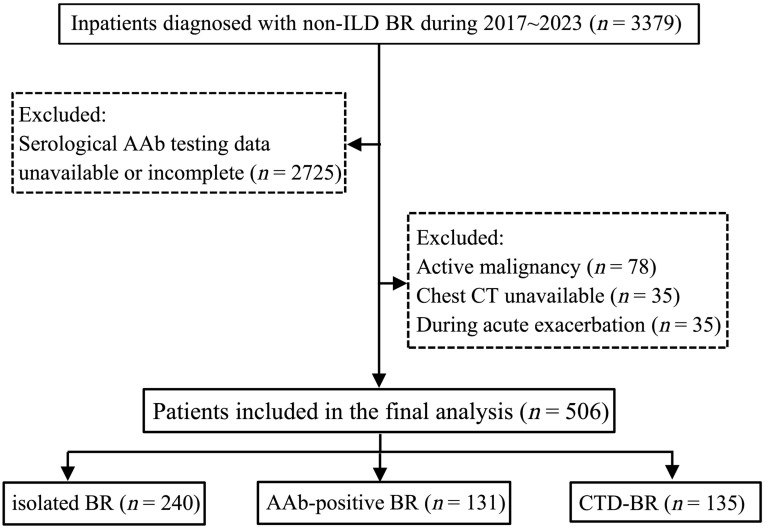
Flow diagram showing patient identification for the study. ILD, interstitial lung disease; BR, bronchiectasis; AAb, autoantibody; CTD, connective tissue disease.

Between 2017 and 2023, 3379 consecutive inpatients with non-ILD BR were screened, of whom 654 had complete serological AAb data. After exclusions, 506 patients with BR were included and stratified into three groups: isolated BR (*n* = 240), AAb-positive BR (*n* = 131), and CTD-BR (*n* = 135). The CTD-BR group consisted of 84 patients with RA and 51 patients with other CTDs, including 27 with AAV, 14 with primary Sjögren’s syndrome, 8 with systemic lupus erythematosus, and 2 with systemic sclerosis. A detailed flowchart is presented in Figure 1. Baseline characteristics did not differ significantly between screened and included patients (Table S1).

Among patients with isolated BR, idiopathic causes were most common (101, 42.1%), followed by post-infective aetiology (92, 38.3%), chronic obstructive pulmonary disease (COPD) (13, 5.4%), and allergic bronchopulmonary aspergillosis (ABPA) (13, 5.4%). In AAb-positive BR, post-infective aetiology predominated (54, 41.2%), followed by idiopathic causes (46, 35.1%), chronic obstructive pulmonary disease (10, 7.6%), and asthma (6, 4.6%). No significant difference was observed between the two groups (Table S2).

### 3.2. Demographics and Clinical Manifestations

The average age of all the patients was 62.4 years, with a mean body mass index (BMI) of 22.6 kg/m^2^. 192 (37.9%) were male, and 126 (24.9%) had a history of smoking. COPD was the most common comorbidity, affecting 187 (37.0%) patients. Asthma, ABPA, chronic pulmonary aspergillosis (CPA), and a history of PTB were observed in 70 (13.8%), 19 (3.8%), 17 (3.4%), and 78 (15.4%) patients, respectively. Cough was the most common respiratory symptom, reported in 451 patients (89.1%), followed by dyspnoea in 320 (63.2%), and haemoptysis in 176 (34.8%) patients. Fever, Raynaud phenomenon, and multiple joint pain were present in 81 (16.0%), 23 (4.5%), and 71 (14.0%) patients, respectively. The median number of acute exacerbations (AE) in the past year was 1.0 (IQR, 0–2.0), and the BSI score was 10.0 (IQR, 7.0–13.0). Regarding treatment, 194 patients (38.3%) were receiving inhaled bronchodilators, 29 (5.7%) were receiving oral corticosteroids (CS), 76 (15.0%) were receiving disease-modifying antirheumatic drugs (DMARDs), and 30 (5.9%) were receiving long-term antibiotic therapy.

Group comparisons revealed that patients with CTD-BR and AAb-positive BR were significantly older than those with isolated BR (mean age 65.8 and 63.9 years vs. 59.6 years, *p* < 0.001). The proportions of male patients and smokers were lower in the CTD-BR group compared with both the isolated BR group (22.5% vs. 46.3%, *p* < 0.001; 15.6% vs. 27.9%, *p* = 0.008) and the AAb-positive BR group (22.5% vs. 39.7%, *p* < 0.001; 15.6% vs. 29.0%, *p* = 0.012). The prevalence of comorbidities was similar across the groups, except for a history of PTB, which was more frequently observed in the CTD-BR and AAb-positive BR groups than the isolated BR group (21.5% and 20.6% vs. 9.2%, *p* < 0.001). Patients with Raynaud phenomenon and multiple joint pain, as well as the use of oral CS and DMARDs, were significantly more prevalent in the CTD-BR group than in the other two groups. In contrast, no significant differences were observed among the three groups for respiratory symptoms, fever, the number of AE in the past year, or BSI scores. The use of inhaled bronchodilators and long-term antibiotic therapy was comparable across groups (Table 1).

### 3.3. Autoantibody Profiles

In the overall population, 12.6% of patients exhibited high-titre ANA. High-titer RF and positive anti-CCP antibodies were detected in 18.6% and 14.8% of patients, respectively. Anti-MPO and anti-PR3 antibodies were positive in 5.5% and 0.2% of patients. Among the ENAs, anti-Ro (SS-A) was the most frequently detected antibody (16.2%), followed by anti-snRNP (7.3%) and anti-La (SS-B) (6.7%).

In the AAb-positive BR group, the most prevalent AAb was anti-Ro (SS-A) (34.4%), followed by anti-snRNP (22.9%), high-titer ANA (21.4%), anti-La (SS-B) (16.8%), and anti-Smith (16.0%). In contrast, the CTD-BR group presented a distinct autoantibody profile, with particularly high frequencies of high-titer RF (52.6%), anti-CCP (54.1%), anti-Ro (SS-A) (27.4%), high-titer ANA (26.7%), anti-MPO (18.5%), and anti-La (SS-B) (8.9%). Statistical differences were observed in the frequencies of high-titer RF, anti-CCP, anti-MPO, anti-snRNP, and anti-Smith antibodies between the two groups (Table 2).

### 3.4. Microorganisms Detected in the Lower Respiratory Tract

Microbiological data from the LRT were available for 464 patients, with 308 samples obtained from sputum and 156 from bronchoalveolar lavage fluid (BALF). Among these, 366 results were derived from culture-based methods, and 98 from targeted next-generation sequencing (tNGS). *Pseudomonas aeruginosa* was the most prevalent pathogen, detected in 26.1% of patients, followed by *Aspergillus* species (6.5%) and *Nocardia* species (3.2%). No statistically significant differences were observed in the prevalence of each pathogen among the isolated BR, AAb-positive BR, and CTD-BR groups, except for a marginal difference in the prevalence of *P. aeruginosa*, which was 22.2%, 25.2%, and 34.2% in the respective groups (*p* = 0.054) (Table 3).

**Figure 2:**
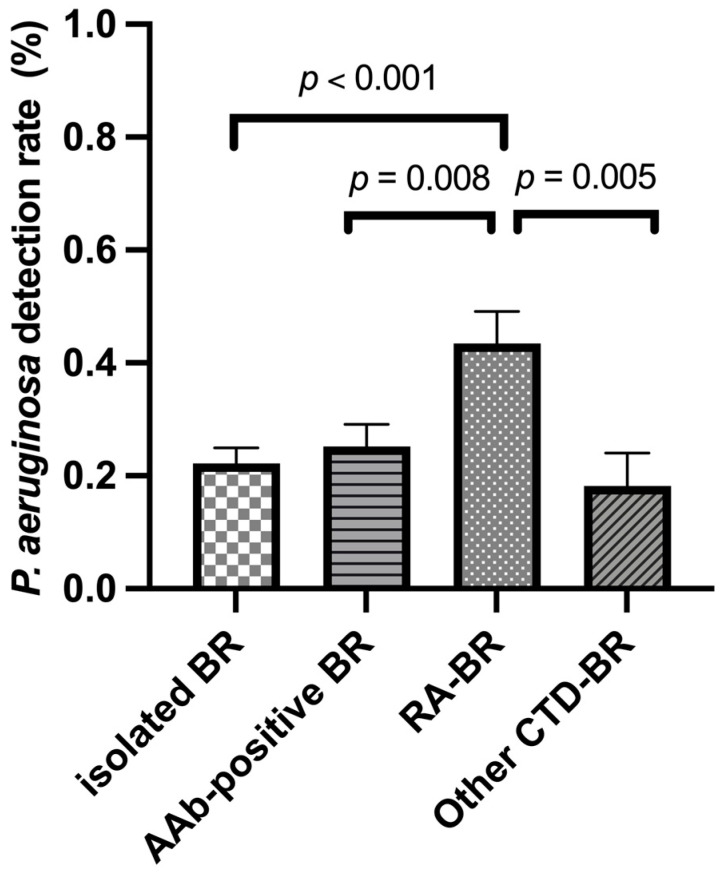
Detection rates of *Pseudomonas aeruginosa* among patients with isolated bronchiectasis (BR), autoantibody (AAb)-positive BR, rheumatoid arthritis (RA) BR, and other connective tissue disease (CTD) BR.

When CTD-BR was further subdivided into RA-BR and other CTD-BR, the *P. aeruginosa* detection rate in RA-BR was significantly higher than in isolated BR, AAb-positive BR, and other CTD-BR (43.4% vs. 22.2%, 25.2%, and 18.2%, *p* = 0.002) (Figure 2). Binary logistic regression analysis revealed that, after adjusting for age, sex, smoking history, BMI, PTB history, BSI score, CS and/or DMARD treatment, FEV1% predicted, and the number of involved lobes, *P. aeruginosa* was independently associated with RA (OR 4.386, 95% CI 1.729–15.328, *p* = 0.007) (Table S3).

### 3.5. Radiographic and Spirometry Features

Among all patients, the lower lobe was most frequently affected (81.2%), followed by the right middle lobe (RML) and lingula (78.9%), and the upper lobe (52.4%). The median number of involved lobes was 3 (IQR, 2–5). Compared with isolated and AAb-positive BR, CTD-BR was associated with a higher prevalence of lower lobe involvement (92.4% vs. 77.1% and 77.0%, *p* < 0.001). Additionally, CTD-BR had a higher median number of involved lobes than isolated BR (4 vs. 3, *p* < 0.001). Pulmonary function testing was conducted in 247 patients (48.8%). The mean values for FEV1% predicted, FVC% predicted, FEV1/FVC ratio, and DLCO_SB_% predicted were 71.6%, 88.8%, 66.0%, and 77.6%, respectively. No statistically significant differences in spirometric indices were observed among the groups (Table 4).

### 3.6. Risk Factors for AAb Positivity and CTD

Variables showing significant differences in univariable analyses (*p* < 0.05) and other key clinical factors were entered into the multinomial logistic regression model. Through analysis, independent risk factors associated with AAb-positive BR (compared with isolated BR) included age (OR 1.062, 95% CI 1.022–1.103, *p* = 0.002) and a history of PTB (OR 3.481, 95% CI 1.239–9.779, *p* = 0.018). For CTD-BR, independent risk factors included age (OR 1.086, 95% CI 1.033–1.141, *p* = 0.001), female sex (OR 2.677, 95% CI 1.021–5.586, *p* = 0.046), a history of PTB (OR 2.011, 95% CI 1.107–6.985, *p* = 0.029), and lower lobe involvement (OR 4.115, 95% CI 1.947–13.672, *p* = 0.015) (Table 5).

## 4. Discussion

This study focused on AAb positivity in patients with BR without ILDs. Clinical data were compared among patients with isolated BR, AAb-positive BR, and CTD-BR. Compared with isolated BR, AAb positivity was mainly associated with ageing and prior PTB and appeared to reflect immune dysregulation rather than a different clinical phenotype, whereas CTD-BR showed more distinct clinical features. A potential link between *P. aeruginosa* and RA was also observed.

In the current study, older age and a history of PTB were identified as risk factors for AAb positivity in patients with BR. Previous epidemiological studies have also reported an age-related increase in AAb positivity [14], possibly due to immunosenescence, a critical driver of autoimmune diseases [15]. In addition to age, a history of PTB was associated with AAb positivity in our study. Increased titres of various autoantibodies have been reported in active TB [16,17]. A prospective analysis in PTB patients further demonstrated elevated RF and other autoimmunity-related markers, along with alterations in B cell and follicular helper T cell subsets, suggesting that *M. tuberculosis* infection is associated with immune dysregulation and features characteristic of autoimmune responses [18]. According to previous reports, autoantibodies may also emerge during TB treatment, although the underlying mechanisms remain unclear [19]. Compared with AAb-positive BR, the relationship between CTD-BR and PTB history may be more complex. On the one hand, infection-triggered AAb production may, over time, contribute to the development of overt CTDs. On the other hand, CTD patients are at high risk of TB infection due to immune disorders and immunosuppressive therapies [20]. Notably, in our study, the AAb spectrum observed in AAb-positive BR was distinct from and less specific than that in CTD-BR, suggesting a state of immune dysregulation rather than a fully developed CTD phenotype.

Apart from older age and prior PTB, AAb-positive BR did not exhibit distinct clinical features compared with isolated BR. In contrast, CTD-related BR presented more extensive lung involvement, particularly with a predominance in the lower lobes and a trend toward a greater number of affected lobes. This lower-lobe predominance may be related to greater perfusion in these regions, which increases exposure to circulating immune complexes and promotes localised inflammatory responses, thereby contributing to airway damage. Taken together, these findings suggest that the presence of autoantibodies alone may have limited clinical significance in BR, whereas established CTD appears to play a more substantial role in shaping disease phenotype. However, whether AAb-positive BR may progress over time to CTD-BR with corresponding clinical features remains unclear, and longitudinal studies are therefore warranted.

One incidental finding of this study was the observed association between *P. aeruginosa* and RA. A previous retrospective study also reported a higher isolation rate of *P. aeruginosa* in the LRT of RA patients compared with non-RA individuals, although BR cases were not specifically distinguished [21]. While immunosuppressive therapy in patients with CTDs may increase susceptibility to *P. aeruginosa* infection, the significantly lower detection rate in other CTDs in our patients suggests that this association may not be fully explained by immunosuppression alone, and that additional disease- or pathogen-specific mechanisms may be involved. Recent studies have highlighted the potential role of *Porphyromonas gingivalis* in RA pathogenesis, particularly through its epidemiological association with periodontal disease and its ability to induce autoimmunity [22,23]. By analogy, we cautiously speculate that certain antigens of *P. aeruginosa* may exhibit molecular mimicry with RA-related autoantigens. Alternatively, persistent colonisation of *P. aeruginosa* may disrupt the ecological and immunological balance between the host and microbiota, promoting the production of RA-related autoantibodies and contributing to RA-associated autoimmunity. Further studies are warranted to validate this association and clarify the underlying mechanisms.

The current study has several limitations. First, as a retrospective single-centre study, selection bias may be present. Only a subset of the initially screened population was included, largely influenced by the clinical decision to perform autoantibody testing, which was more likely in patients suspected of having CTDs. This may have resulted in an overrepresentation of AAb-positive cases and limited the generalisability of our findings. Second, the retrospective and observational nature of the study precludes causal inference. The associations observed between AAb positivity and post-TB status, as well as between *P. aeruginosa* infection and RA, may be influenced by residual confounding and should therefore be interpreted with caution. Third, longitudinal follow-up data on acute exacerbations were not consistently available and were therefore not included as an outcome. Future prospective multicentre studies are warranted to validate these findings and clarify the potential pathogenic link between airway infection and autoimmunity.

In conclusion, our study demonstrated that in BR patients without ILDs, AAb positivity presented limited clinical differences compared with isolated BR, with older age and prior PTB independently associated, possibly reflecting underlying immune dysregulation. RA-BR showed a potential independent association with *P. aeruginosa* isolation, warranting further investigation.

## Figures and Tables

**Table 1:** Demographics and clinical manifestations of the study population.

	All	Isolated BR	AAb-Positive BR	CTD-BR	*p* Value
*N*	506	240	131	135	
Age, years	62.4 ± 12.6	59.6 ± 13.5	63.9 ± 12.5 *	65.8 ± 9.5 *	<0.001
Male	192 (37.9)	111 (46.3)	52 (39.7)	29 (22.5) *^#^	<0.001
BMI, kg/m^2^	22.6 ± 4.2	22.5 ± 4.0	22.8 ± 4.0	22.8 ± 4.6	0.765
Smoking history	126 (24.9)	67 (27.9)	38 (29.0)	21 (15.6) *^#^	0.013
Comorbidities					
COPD	187 (37.0)	94 (39.2)	53 (40.5)	40 (29.6)	0.119
Asthma	70 (13.8)	35 (14.6)	20 (15.3)	15 (11.1)	0.560
ABPA	19 (3.8)	12 (5.0)	6 (4.6)	1 (0.7)	0.103
CPA	17 (3.4)	9 (3.8)	5 (3.8)	3 (2.2)	0.735
History of PTB	78 (15.4)	22 (9.2)	27 (20.6) *	29 (21.5) *	<0.001
Symptoms					
Cough	451 (89.1)	210 (87.5)	117 (89.3)	124 (91.9)	0.425
Dyspnoea	320 (63.2)	147 (61.3)	86 (65.6)	87 (64.4)	0.669
Haemoptysis	176 (34.8)	91 (37.9)	43 (32.8)	42 (31.1)	0.354
Fever	81 (16.0)	33 (13.8)	19 (14.5)	29 (21.5)	0.128
Raynaud phenomenon	23 (4.5)	0 (0)	2 (1.5)	21 (15.5) *^#^	<0.001
Multiple joints pain	71 (14.0)	2 (0.8)	6 (4.6)	63 (46.7) *^#^	<0.001
Number of AE in the past year	1.0 (0–2.0)	1.0 (0–1.0)	1.0 (0–1.0)	1.0 (0–2.0)	0.275
BSI score	10.0 (7.0–13.0)	9.0 (7.5–11.0)	10.0 (6.0–14.0)	12.0 (8.0–15.0)	0.046
Treatments					
Inhaled bronchodilators	194 (38.3)	88 (36.6)	53 (40.4)	53 (39.3)	0.752
Oral corticosteroids	29 (5.7)	0 (0)	0 (0)	29 (21.5) *^#^	<0.001
DMARDs	76 (15.0)	0 (0)	0 (0)	76 (56.3) *^#^	<0.001
Long-term antibiotics	30 (5.9)	12 (5.0)	9 (6.9)	9 (6.7)	0.736

Values were given as mean ± SD, median (IQR), or *n* (%). * *p* < 0.017 versus the isolated BR group, ^#^ *p* < 0.017 versus the AAb-positive BR group. BR: bronchiectasis; AAb: autoantibody; CTD: connective tissue disease; BMI: body mass index; COPD: chronic obstructive pulmonary disease; ABPA: allergic bronchopulmonary aspergillosis; CPA: chronic pulmonary aspergillosis; PTB: pulmonary tuberculosis; AE: acute exacerbation; BSI: Bronchiectasis Severity Index; DMARDs: disease-modifying antirheumatic drugs.

**Table 2:** Autoantibody profiles of the study population.

Positive Antibodies	AAb-Positive BR	CTD-BR	*p* Value
*N*	131	135	
High-titer ANA (≥1:320)	28 (21.4)	36 (26.7)	0.320
RF titer ≥ 2 × ULN	23 (17.6)	71 (52.6)	<0.001
Anti-CCP	2 (1.5)	73 (54.1)	<0.001
Anti-myeloperoxidase	3 (2.3)	25 (18.5)	<0.001
Anti-proteinase3	1 (0.8)	0 (0)	0.492
Anti-Ro (SS-A)	45 (34.4)	37 (27.4)	0.234
Anti-La (SS-B)	22 (16.8)	12 (8.9)	0.066
Anti-ribonucleoprotein	30 (22.9)	7 (5.1)	<0.001
Anti-Smith	21 (16.0)	6 (4.4)	0.002
Anti-centromere	6 (4.6)	2 (1.5)	0.168
Anti-ribosomal P protein	6 (4.6)	1 (0.7)	0.063
Anti-histone	5 (3.8)	1 (0.7)	0.116
Anti-nucleosome	5 (3.8)	2 (1.5)	0.276
Anti-dsDNA	4 (3.1)	1 (0.7)	0.208
Anti-Jo-1	3 (2.3)	0 (0)	0.118
Anti-Scl-70	2 (1.5)	1 (0.7)	0.618

Values were given as the *n* (%). BR: bronchiectasis; AAb: autoantibody; CTD: connective tissue disease; ANA: antinuclear antibody; RF: rheumatoid factor; ULN: upper limit of normal; CCP: anti-cyclic citrullinated peptide.

**Table 3:** Microorganisms detected in the lower respiratory tract.

Microbiology	All	Isolated BR	AAb-Positive BR	CTD-BR	*p* Value
*N* (%)	464 (91.7)	221 (92.1)	123 (93.9)	120 (88.9)	0.337
Source of microbiology					
Sputum/BALF (*N*1/*N*2)	308/156	136/85	85/38	87/33	0.091
Culture/tNGS (*N*1/*N*2)	366/98	165/56	102/21	99/21	0.106
Positive results					
*Pseudomonas aeruginosa*	121 (26.1)	49 (22.2)	31 (25.2)	41 (34.2)	0.054
*Aspergillus* species	30 (6.5)	15 (6.8)	10 (8.1)	5 (4.2)	0.450
*Nocardia* species	15 (3.2)	10 (4.5)	4 (3.3)	1 (0.8)	0.188
Nontuberculosis *mycobacteria*	14 (3.0)	10 (4.5)	2 (1.6)	2 (1.7)	0.279
*Klebsiella pneumoniae*	12 (2.6)	7 (3.2)	1 (0.8)	4 (3.3)	0.357
*Mycobacterium tuberculosis*	11 (2.4)	8 (3.6)	2 (1.6)	1 (0.8)	0.301
*Haemophilus influenzae*	9 (1.9)	4 (1.8)	2 (1.6)	3 (2.5)	0.829
*Staphylococcus aureus*	4 (0.9)	2 (0.9)	0 (0)	2 (1.7)	0.284
Others	23 (5.0)	13 (5.9)	5 (4.1)	5 (4.2)	0.731

Values were given as the *n* (%) or N1/N2. BR: bronchiectasis; AAb: autoantibody; CTD: connective tissue disease; BALF: bronchoalveolar lavage fluid; tNGS: targeted next-generation sequencing.

**Table 4:** Radiographic and spirometry features of the study population.

	All	Isolated BR	AAb-Positive BR	CTD-BR	*p* Value
*N*	506	240	131	135	
Relevant lobe involvement					
Upper lobe	259 (47.6)	114 (48.3)	70 (55.6)	75 (56.8)	0.212
RML + lingual	390 (78.9)	178 (75.4)	106 (84.1)	106 (80.2)	0.138
Lower lobe	401 (81.2)	182 (77.1)	97 (77.0)	122 (92.4) *^#^	<0.001
Number of involved lobes	3 (2–5)	3 (2–4)	3.5 (2–5)	4 (2–5) *	0.005
Spirometry	247 (48.8)	123 (51.2)	65 (49.6)	59 (43.7)	0.371
FEV1, % predicted	71.6 ± 29.1	74.4 ± 29.1	69.0 ± 30.4	65.3 ± 26.7	0.107
FVC, % predicted	88.8 ± 24.8	91.2 ± 25.1	86.6 ± 25.9	85.4 ± 22.5	0.237
FEV1/FVC, %	66.0 ± 14.4	64.8 ± 13.6	62.0 ± 16.4	61.0 ± 13.7	0.186
DLCO_SB_, % predicted	77.6 ± 19.6	78. 5 ± 20.6	74.2 ± 20.7	72.7 ± 14.8	0.146

Values were given as mean ± SD, median (IQR), or *n* (%). * *p* < 0.017 versus the isolated BR group, ^#^ *p* < 0.017 versus the AAb-positive BR group. BR: bronchiectasis; AAb: autoantibody; CTD: connective tissue disease; RML: right middle lobe; FEV1: forced expiratory volume in the first second; FVC: forced vital capacity; DLCO_SB_: single-breath diffusing capacity of the lung for carbon monoxide.

**Table 5:** Multinomial logistic regression analysis for factors related to AAb positivity and CTD.

	AAb-Positive BR vs. Isolated BR		CTD-BR vs. Isolated BR	
Covariates	OR (95% CI)	*p* Value	OR (95% CI)	*p* Value
Age (years)	1.062 (1.022–1.103)	0.002	1.086 (1.033–1.141)	0.001
Sex (female)	1.335 (0.460–3.874)	0.595	2.677 (1.021–5.586)	0.046
Smoking history (yes)	1.644 (0.502–5.379)	0.411	2.642 (0.615–9.349)	0.191
History of PTB (yes)	3.481 (1.239–9.779)	0.018	2.011 (1.107–6.985)	0.029
BSI score	0.919 (0.801–1.054)	0.225	1.018 (0.865–1.198)	0.831
FEV1, % predicted	0.989 (0.972–1.005)	0.165	0.993 (0.974–1.011)	0.368
Number of involved lobes	0.868 (0.641–1.177)	0.362	0.853 (0.603–1.206)	0.368
Lower lobe involvement (yes)	2.634 (0.887–7.826)	0.101	4.115 (1.947–13.672)	0.015

AAb: autoantibody; BR: bronchiectasis; CTD: connective tissue disease; OR: odds ratio; PTB: pulmonary tuberculosis; BSI: Bronchiectasis Severity Index; FEV1: forced expiratory volume in the first second.

## Data Availability

The data used and analysed in this study are included in the article or are available upon reasonable request.

## Supplementary Materials

The following supporting information can be downloaded at https://www.mdpi.com/article/10.3390/jcm15082897/s1. Table S1: Baseline characteristics of the included patients compared with the entire screened population; Table S2: Underlying causes of BR in isolated and AAb-positive BR groups; Table S3. Binary logistic regression analysis for factors related to RA in BR patients.
