# Supplementary material for: Autoantibody Positivity in Bronchiectasis Without Interstitial Lung Diseases: Risk Factors and Impact on Clinical Features
Source: J Clin Med. 2026 Apr 10;15(8):2897. doi: 10.3390/jcm15082897 (PMC13116931; doi:10.3390/jcm15082897)
Supplement: Supplementary file 1 [file jcm-15-02897-s001.zip › jcm-4190133-supplementary.pdf]

Table S1. Baseline characteristics of the included patients compared with the entire screened population.

	**Patients screened**	**Patients included**	***p* value**
N	3379	506	
Age, years	63.1 ± 13.2	62.4 ± 12.6	0.256
Male	1365 (40.4)	192 (37.9)	0.307
BMI, kg/m2	22.3 ± 4.9	22.6 ± 4.2	0.723
Smoking history	902 (26.7)	126 (24.9)	0.418
Comorbidities			
COPD	1230 (36.4)	187 (37.0)	0.843
Asthma	418 (12.4)	70 (13.8)	0.388
ABPA	92 (2.7)	19 (3.8)	0.197
CPA	87 (2.6)	17 (3.4)	0.375
History of PTB	421 (12.5)	78 (15.4)	0.064

Values were given as the mean ± SD or *n* (%).

BMI: body mass index; COPD: chronic obstructive pulmonary disease; ABPA: allergic bronchopulmonary aspergillosis; CPA: chronic pulmonary aspergillosis; PTB: pulmonary tuberculosis.

Table S2. Underlying causes of BR in isolated and AAb-positive BR groups.

Etiology	Isolated BR	AAb-positive BR	*p* value
N	240	131	
Idiopathic	101 (42.1%)	46 (35.1%)	0.222
Post-infective	92 (38.3%)	54 (41.2%)	0.657
COPD	13 (5.4%)	10 (7.6%)	0.500
ABPA	13 (5.4%)	5 (3.8%)	0.617
Asthma	5 (2.1%)	6 (4.6%)	0.206
Ankylosing spondylitis	5 (2.1%)	3 (2.3%)	1.000
GERD	4 (1.7%)	4 (3.1%)	0.460
Inflammatory bowel disease	3 (1.3%)	2 (1.5%)	1.000
Primary ciliary dyskinesia	3 (1.3%)	1 (0.8%)	1.000
Primary immunodeficiency	1 (0.4%)	0 (0)	1.000
Diffuse panbronchiolitis	0 (0)	2 (1.5%)	0.124

Values were given as the *n* (%).

BR: bronchiectasis; AAb: autoantibodies; COPD: chronic obstructive pulmonary disease; ABPA: allergic bronchopulmonary aspergillosis; GERD: gastroesophageal reflux disease.

Table S3. Binary logistic regression analysis for factors related to RA in BR patients.

**Covariates**	**OR (95% CI)**	***p* value**
Age (years)	1.047 (1.006-1.081)	0.045
Sex (female)	0.664 (0.157-2.810)	0.578
Smoking history (yes)	0.455(0.073-2.845)	0.400
BMI (kg/m2)	1.100 (0.973-1.244)	0.129
History of PTB (yes)	1.298 (0.317-5.320)	0.717
BSI score	0.871 (0.707-1.074)	0.197
P. aeruginosa detected in LRT (yes)	4.386 (1.729-15.328)	0.007
CS and/or DMARD treatment	13.560 (3.245-29.107)	<0.001
Number of involved lobes	1.207 (0.823-1.770)	0.335
FEV1, % predicted	0.991 (0.968-1.015)	0.469

RA: rheumatoid arthritis; BR: bronchiectasis; OR: odds ratio; BMI: body mass index; PTB: pulmonary tuberculosis; BSI: Bronchiectasis Severity Index; LRT: lower respiratory tract; CS: corticosteroids; DMARDs: disease-modifying antirheumatic drugs; FEV1: forced expiratory volume in the first second.
